# A Short Review on Nanostructured Carbon Containing Biopolymer Derived Composites for Tissue Engineering Applications

**DOI:** 10.3390/polym15061567

**Published:** 2023-03-21

**Authors:** Mattia Bartoli, Erik Piatti, Alberto Tagliaferro

**Affiliations:** 1 Center for Sustainable Future Technologies (CSFT), Istituto Italiano di Tecnologia (IIT), Via Livorno 60, 10144 Turin, Italy; mattia.bartoli@polito.it; 2 Consorzio Interuniversitario Nazionale per la Scienza e Tecnologia dei Materiali (INSTM), Via G. Giusti 9, 50121 Florence, Italy; 3 Department of Applied Science and Technology, Politecnico di Torino, Corso Duca degli Abruzzi 24, 10129 Turin, Italy; erik.piatti@polito.it; 4 Faculty of Science, Ontario Tech University, 2000 Simcoe Street North, Oshawa, ON L1G 0C5, Canada

**Keywords:** graphene, carbon nanotubes, biopolymers, scaffolds, tissue engineering

## Abstract

The development of new scaffolds and materials for tissue engineering is a wide and open realm of material science. Among solutions, the use of biopolymers represents a particularly interesting area of study due to their great chemical complexity that enables creation of specific molecular architectures. However, biopolymers do not exhibit the properties required for direct application in tissue repair—such as mechanical and electrical properties—but they do show very attractive chemical functionalities which are difficult to produce through in vitro synthesis. The combination of biopolymers with nanostructured carbon fillers could represent a robust solution to enhance composite properties, producing composites with new and unique features, particularly relating to electronic conduction. In this paper, we provide a review of the field of carbonaceous nanostructure-containing biopolymer composites, limiting our investigation to tissue-engineering applications, and providing a complete overview of the recent and most outstanding achievements.

## 1. Introduction

Complex chemical architectures represent a challenge for material science and synthetic chemistry. Several approaches can be used to achieve the complexity that naturally occurs in living organisms during anabolic metabolism. The level of complexity shown by biopolymers is astonishing and represents an opportunity to apply ready-to- use complex building blocks for the production of new materials [1]. This is particularly interesting for all those applications that require replicable polymeric matrixes with morphology and chemical functionalities that are hard to synthesize in vitro. Among possible applications, tissue repair and engineering is one of the most challenging [2], requiring high biocompatibility and an ability to mimic the original tissue texture. Biopolymers could be successfully used for this application to achieve remarkable results [3,4,5]. Nonetheless, native biopolymers do not exhibit suitable mechanical properties and electrical conductivity, excluding their use for both structural and neuronal/nerve tissue treatments. An elegant solution is represented by mixing neat biopolymers with fillers that are able to improve their mechanical and electrical features [6]. Good candidates for this task are nanostructured carbonaceous materials [7]. Nanostructured carbon is a wide family that comprises several species, from carbon black to the most cutting-edge materials, such as carbon nanotubes (CNTs) and graphene-related materials. These last two are the most remarkable with regard to both electrical and mechanical properties, as described in the recent literature [8,9], though their production is expensive and their dispersion into polymers is still rather challenging and costly. Nevertheless, the great challenge represented by the use of biopolymers in tissue engineering requires solutions beyond the state of the art—coupling with nanostructured carbon is the most promising route to fulfill this task [10,11,12]. In this work, we review the use of both CNTs and graphene-related materials as fillers for biopolymers used in tissue engineering. We lay out a clear picture of the latest achievements in the field, providing an introduction to the main topic before presenting more in-depth discussion. We consider biopolymer composites in two main categories: protein-derived and polysaccharide-based.

## 2. A Brief Overview on the Use of Polymers in Tissue Engineering

**Figure 1:**
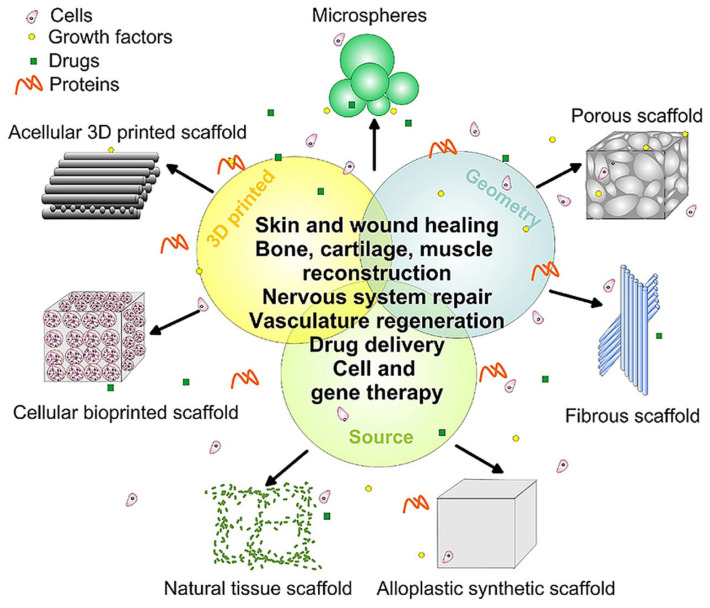
Multiple polymer- and polymer-composite-based routes to tissue engineering, highlighting the main morphologies used. Reprint with permission from Nikolova et al. [13] under CC BY license.

Tissue engineering is one of the most cutting-edge applications of polymer science, aiming to repair and replace damaged biological tissues using polymers and polymer composites by means of several production routes, as illustrated in Figure 1.

Over the last two decades, the realm of tissue engineering underwent rapid developments and several experimental routes for the reparation and regeneration of organs, tissues and cells were developed [2]. Furthermore, the use of the tissue-engineering approach has proved a promising alternative response to pathologies when traditional chemotherapy has failed [14]. Such a promising tool has inspired researchers to invest effort in quickly reaching the highest technological readiness level achievable. Nonetheless, this frontier is still far away due to the complexity of biological systems and their interaction with chemicals. Accordingly, the first experimental efforts were focused on cell implantation or replacement, creating artificial replacements for damaged tissues [15]. Cell-based tissue engineering remains quite complex, whereas the use of biopolymer-formulated scaffolds is cheaper and more tunable. Generally, a polymeric scaffold is defined as an intrinsic porous three-dimensional solid that interacts with cell lines and can be used to repair damage by merging with native tissue. Biopolymers play a crucial role due to their biocompatibility with biological matrixes, promoting the transport of solutions and gases and the proliferation of cell lines [5]. Biopolymer scaffolds are generally biodegradable, are able to match the same rate of regeneration of the native tissue, and enable its replacement with new undamaged tissues without releasing harmful species [16]. The first report of such materials was a 1975 study by Yannas et al. [17] based on the use of native collagen functionalized with glycosaminoglucans, followed by a systematic investigation of its use as a skin repair agent [18]. Biopolymers are also used as suitable replacements for damaged tissues, such as in the use of ocular, bone, vascular and dental implants [19].

The great advantage of biopolymers compared with synthetic polymers is associated with the complexity of their molecular architectures and their reproducibility resulting from biological pathways. The great potential for the application of biologically synthesized polymers, together with the role of purification procedures, is evidenced by considering cellulose as a case study. The structure of cellulose was determined by Staudinger in the early years of the 20th century [20], but only in 2001 was it fully synthesized in vitro [21]. Even now, more complex biopolymers, such as chitin or lignin, cannot be feasibly produced using chemical synthesis. Different considerations apply to protein synthesis, where a solid-state automatized synthetic approach provides new means for the in vitro production of peptides and proteins [22]. Biopolymer use has extended to several fields of the biomedical sciences, from drug delivery {Catania, 2021 #10529} to wound dressing [23].

**Figure 2:**
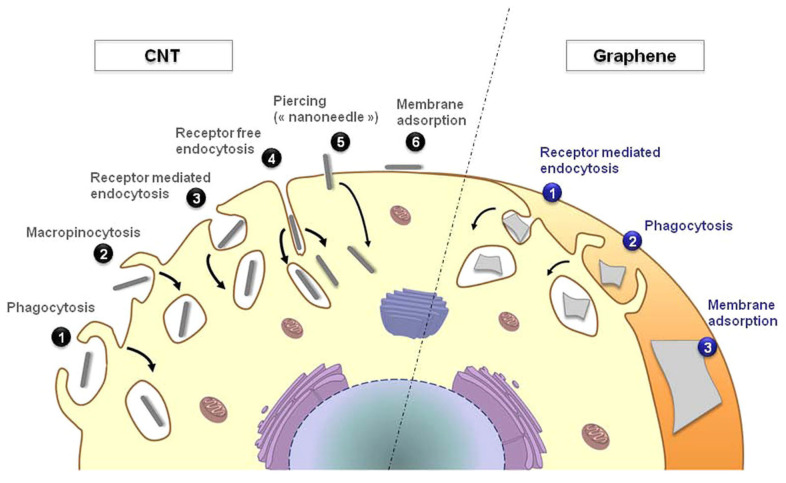
Schematic route of the cellular uptake of both neat CNTs and graphene-related materials. Reprinted with permission from Bussy et al. [27] (Copyright 2012 American Chemical Society).

Nevertheless, several biological applications—ranging from scaffolding to prosthetics—require superior conductive and mechanical properties. Neat biopolymers do not perform well enough to be used without mixing them with fillers. Nanostructured fillers, such as graphene derivatives [24] and CNTs [25], have found several applications as fillers for the production of scaffolds and prosthetics. Nevertheless, the interaction between the cellular structure and both CNTs and graphene-like materials is a very complex topic, as shown in Figure 2 [26]. All the interaction mechanisms shown are of major importance for the design of carbon-based biocompatible/bioactive composites [12] and are discussed in the next sections.

## 3. Nanostructured Carbon Materials

### 3.1. Graphene and Graphene-Like Materials

**Figure 3:**
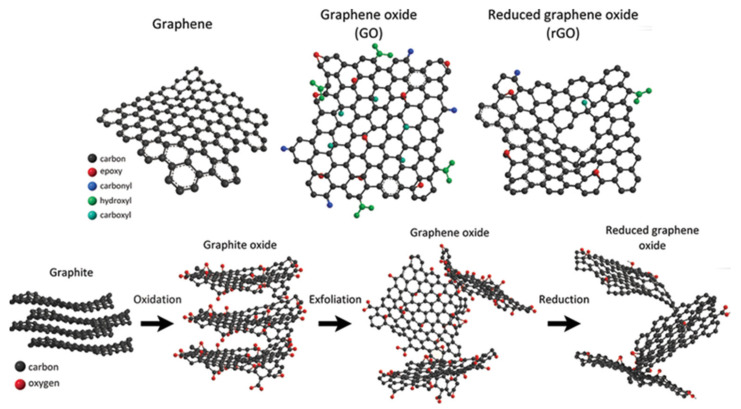
Methodologies for the production of graphene and related material structures through the oxidation of graphite. Reprinted from Jimenez-Cervantes et al. [28] under CC BY license.

Graphene, graphene oxide (GO) and reduced GO (rGO) are briefly illustrated in Figure 3.

In its ideal form, graphene is a two-dimensional crystal of carbon atoms bound in a honeycomb lattice and can be thought as a single plane of graphite. In each elementary cell of graphene, each carbon atom is linked by three in-plane σ bonds to its neighboring atoms with sp^2^ hybridization, while the p orbitals perpendicular to the sp^2^ plane do not participate in the bonding, hosting the conduction electrons. As a consequence, the electrons in the π bond are delocalized over the entire lattice structure and are able to move freely in the graphene plane [29,30,31], enabling high electrical conductivity despite the monolayer nature of the material [4]. The peculiar structure of graphene gives rise to unique electronic properties, including low-energy electrons behaving as massless Dirac fermions, ultra-high carrier mobilities up to $∼10^{6}$ cm^2^ V^−1^ s^−1^, room-temperature ballistic transport and half-integer quantum Hall effects [32]. These features have made graphene extremely appealing for high-frequency electronics and optoelectronics, while its ultrahigh tensile strength and flexibility make it very well suited for being incorporated in flexible devices [33].

The astonishing properties exhibited by ideal graphene are counterbalanced by its poor compatibility with established technological pipelines, as well as its scarce availability [34]. Indeed, the term graphene is often used in the literature to refer to other related carbon allotropes, including few-layer graphene and nanographite [33,35,36,37]. Several different materials have been proposed as alternatives that are able to overcome the issues associated with the use of “real” graphene. A first alternative is GO (graphene oxide), which is an oxidized graphene compound rich in oxygen functionalities [38]. Among these, epoxide and hydroxyl residual groups are predominant on the GO basal plane, whereas carbonyl and carboxylic groups are mostly localized along the edges. However, while the structure of pure graphene is relatively robust against different production methods, the structure of GO is deeply affected by its production process.

Due to this sensitivity to the production path, several structures have been proposed for GO in different studies, such as the Hofmann, Ruess, Scholz–Boehm, Nakajima–Matsuo, Lerf–Klinowski, and Szabo models [39,40].

Among these, the Lerf–Klinowski model is usually considered to represent the most realistic description of actual GO [16], attributing the presence of defects in the final material—such as holes, wrinkles, and cracks—mostly to the oxidation process.

GO is not the only graphene-derived material currently under extensive scientific investigation. Another very promising graphene-based material is reduced graphene oxide (rGO), which is obtained through a reduction process of GO by means of several reducing agents [41]. This process is necessary to eliminate the oxygenated functional groups of GO, which improve its handleability, but also result in an almost complete suppression of the high conductivity of neat graphene. The different production paths of GO result in different profiles of oxygen-containing functionalities and deeply alter the final properties of rGO [42]. High electrical conductivities are usually achieved in rGO, which is usually formed with oxygen amounts of between 1.5 and 0.4 wt.% [43].

Despite their astonishing properties, graphene-related materials can damage cellular structures, promoting oxidative degradation, apoptosis and cell-membrane disruption [27], mainly mediated by oxidation induced by peroxidase [44]. Modelling and tuning the interactions between graphene-like materials and cells is a challenging task and requires very good knowledge of the graphene-like flake sizes and thicknesses [45] as well as their functionalities [46].

The use of graphene and related materials for biological applications can exploit several interesting features [47,48]. The addition of GO or rGO to several polymeric matrixes has been reported to be effective for the replacement of damaged tissues through surface interactions [49]. The immobilization of graphene-related materials inside a polymeric host avoids the immunogenic [50] and inflammatory [51] effects they promote after cellular uptake.

As reported by Bahrami et al. [52], graphene flakes dispersed into polyurethane promote the growth of fibroblasts and endothelial cells on membranes containing graphene flakes. The findings were particularly interesting regarding endothelial cells. These could be grown on the inner surface of tubular scaffolds by mimicking the native blood vessel structure. The authors of the study demonstrated the ability of graphene-based composites to support the attachment, spreading and proliferation of cell lines. A similar approach was used by Pant et al. [53] for the production of coated stents using graphene oxide mixed with polyurethane.

### 3.2. Carbon Nanotubes

**Figure 4:**
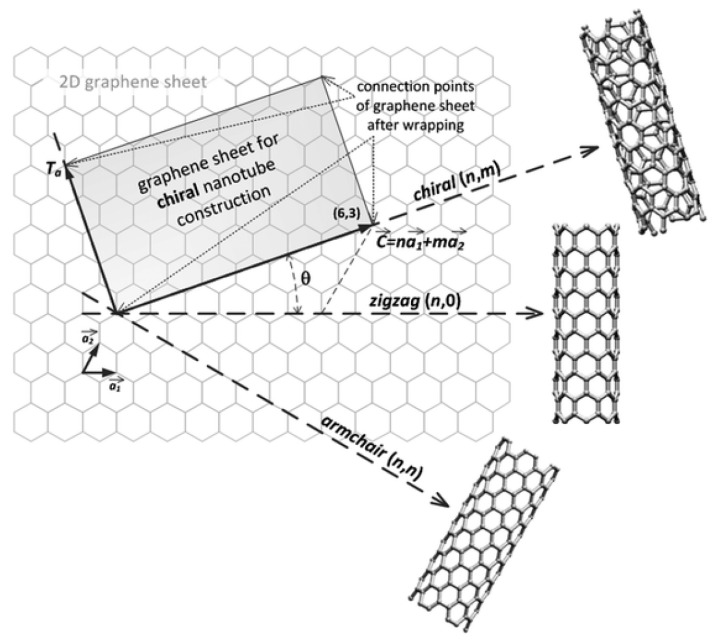
Theoretical folding of a perfect graphene sheet according to different linear combinations of the chiral vectors, leading to the three main CNT structures, named armchair, zig-zag and chiral. Reprinted with permission from Sanginario et al. [55] under CC BY license.

CNTs are another carbon allotrope based on the honeycomb lattice of sp^2^-hybridized carbon atoms and can be thought of as cylindrical graphite sheets rolled up in a tubular structure. Different types of folding are possible for CNTs according to the so-called chiral vector $\vec{C}=n\;{\vec{a}}_{1}+m\;{\vec{a}}_{2}$, where *n* and *m* are the positive integer chiral indexes, and ${\vec{a}}_{1},\;{\vec{a}}_{2}$ are the graphene in-plane lattice vectors. As shown in Figure 4, the chiral indexes enable identification of three different orientations of the longitudinal axis of the nanotube with respect to the hexagonal lattice of graphene, resulting in three different CNT types termed zig-zag (m=0), armchair (m=n), and chiral (n≠m≠0). Chiral indexes have also been shown to enable prediction of whether the electronic properties of CNTs are metallic or semiconducting [54]; specifically, if $\left|n-m\right|=3q$ with n≠m (where *q* is a positive integer) the CNT is metallic, otherwise it is semiconducting. Armchair CNTs always show metallic behavior, whereas chiral CNTs exhibit the same geometric properties as enantiomeric molecules.

Furthermore, CNT models can be obtained by folding both single-layer and multi-layer graphene sheets, resulting in single-walled CNTs (SWCNTs) and multi-walled CNTs (MWCNTs), respectively, even if real CNT structure is more complex [56]. This choice affects the structural properties of CNTs, with SWCNTs exhibiting diameters ranging between 3 Å and 1 nm [57], whereas the diameter of MWCNTs can exceed 100 nm [58,59]. The CNT length, on the other hand, is highly dependent on the production process for both types and can range from a few nanometers up to a few tens of cm [60,61,62]. The extremities of CNTs can either be open or closed with fullerene-type caps [30] and variously functionalized [63]. Similar to graphene, the sp^2^-hybridized honeycomb carbon lattice endows CNTs with excellent electrical [64,65], mechanical [66] and thermal [67,68] properties.

Similarly to graphene-like materials, CNTs are harmful for tissues and cells due to intrinsic defects [69,70] and metallic catalyst impurities [70,71], slowing down their use for neuronal tissue repair where their electric conductivity could otherwise enable game-changing approaches. The cytotoxicity and harmful effects of CNTs may be explained by the “*pathogenic fiber paradigm*” model which states that thin, long and persistent fibrous structures promote both inflammation and carcinogenesis [72]. Nevertheless, the real effects of CNT radical activity is still controversial, as shown by the scavenging effect reported by Fenoglio et al. [73]. After incorporation into a polymeric matrix, CNT toxicity can be minimized, as already reported for graphene-related materials [74,75]. This has enabled their use for the production of membranes with low filler loading (up to 0.5 wt.%) for neuronal tissue repair [76]. Furthermore, CNT-filled scaffolds can be used effectively for electrical stimulation to promote neurite overgrowth [77].

## 4. Carbon-Containing Biopolymer Composites for Biomedical Applications: An Overview

### 4.1. Nanostructured Carbon-Containing Proteinaceous Composites

#### 4.1.1. Collagen

Collagen is a fibrous protein exhibiting strong hydrophilic behavior, which is insoluble in the majority of organic solvents, and represents the most abundant component of connective tissue. Remarkably, collagen accounts for more than 30 wt.% of total animal proteins, representing the main component of the extracellular matrix in animals. Collagen shows very interesting biological activities, such as the inhibition of tissue damage induced by stretching stress [78], preserving the structure of cartilage, bones and blood vessels.

The medical use of collagen started between the end of the 19th and the beginning of the 20th century with the development of biodegradable sutures for intestine surgery [79]. Neat collagen is widely used as a replacement for repairing corneal tissue due to its high bioadhesivity, biocompatibility with reduced immunogenicity, and acceptable mechanical properties [80]. Additionally, collagen fibrils can pass through epithelial tissue of the cornea stacking themselves onto native collagen [81]. In addition, collagen promotes the cellular adhesion, proliferation and differentiation of multiple cellular lines, promoting enhanced regeneration of several tissues [82].

The complex mechanisms of collagen degradation are central issues in the use of neat collagen associated with its high hydrophilicity that induces denaturation pathways and swelling in in vivo experiments [83]. Agarwal et al. [84] surmounted this issue by entrapping graphene sheets into a highly crosslinked collagen cryogel. The graphene-related composite was studied using X-ray tomography, showing improvement in both porosity and the porous connections of collagen cryogels induced by graphene flakes, together with thermal stability [85]. Nonetheless, the most used filler among the graphene-like materials for the production of composites remains GO, which is able to drive the self-assembly of hydrogels using a loading of up to 4 wt.% [86].

A cryogel based on mixed rGO flakes and collagen has found a promising application in bone restoration, as reported by Bahrami et al. [87]. The authors demonstrated that the mechanical strength of rGO-coated collagen cryogel was enhanced by three times compared with the uncoated cryogel. Furthermore, the immobilized rGO flakes promoted total biocompatibility of the collagen-based scaffold, enhancing mesenchymal stem cell proliferation. The authors also showed the ability of this scaffold to promote bone regeneration in a rabbit animal model.

A simple sol-gel production route is also effective for GO-containing collagen composites, as demonstrated by Liu et al. [88]. The authors produced a hydrophilic scaffold with good bone mineralization by adding 0.1 wt/v% GO to type I collagen during in vitro experiments. The osteogenesis promoted by the GO-based collagen scaffold could be boosted by adding inorganics, such as strontium, to the mixture [89], activating a protein complex responsible for bone-cell proliferation and differentiation. GO-based collagen composites can also be coupled with hydroxyapatite, as reported by Zhou et al. [90]. The authors crafted a multi-layer mineralized composite able to upregulate genes involved in osteogenesis, providing a comfortable microenvironment for hosting the cells of bone tissues. Similar results were obtained using bone apatite [91] due to the ability of the carboxylic residues on GO edges to provide a gripping point for inducing mineralization. These materials may also be used for cartilage repair, as reported by Lyu et al. [92]. The authors demonstrated the ability of GO-based collagen composites to induce the growth of chondrocytes, forming a cartilage matrix through the upregulation of metalloproteinases or their inhibitors. This double regulation enhanced the formation of homogenous cartilage tissue with regular distribution and porosity. A very advanced application of graphene-derivative-based collagen materials is related to the regeneration of neuronal tissue and damaged nerves. As reported by Guo and co-workers [93], rGO-based collagen scaffolds were able to enhance neuronal differentiation due to high conductivity. Agarwal et al. [94] followed similar protocols using amine-tailored graphene flakes dispersed into collagen to match both the electrical conductivity of 3 S/m and the Young’s modulus of around 350 kPa of spinal cord tissue. The authors successfully suppressed the inflammatory response in rats, showing a remarkable ability of the scaffold to avoid macrophage proliferation. A more traditional use of GO-containing collagen composites is represented by using them as a coating for inorganic biocompatible alloys [95], inducing useful antifibrotic [96] and antibacterial [97] effects.

Interestingly, CNTs containing collagen scaffolds are more effective as substrates for the proliferation and differentiation of stem cells [98,99,100] and could be internalized into the cells without inducing disruption of the cellular membrane [101]. CNT-based collagen scaffolds have been used for applications that require very high electrical conductivity together with enhanced stiffness [102,103].

Chi et al. [104] reported the possibility of using CNT-based collagen composites for the stimulation of fibroblasts by simply preparing an electrospun mate. Considering its high customizability, the authors proposed the use of such produced materials for the treatment of dysfunctional fibroblasts associated with cartilaginous disorders. The conductivity of CNT-based collagen composites has been exploited in the production of advanced procedures for the treatment of neural degeneration [105], efficiently promoting neuronal growth and achieving remarkable results in nerve regeneration. The high conductivity of CNT-containing collagen also represents a tool for the production of electrically responsive cardiac patches, though the mechanism of action is still debated [106].

#### 4.1.2. Keratin

Keratin is a protein in which cysteine reaches up to 13 wt.%; it is the major constituent of structures such as feathers and horns. The use of keratin as a biological scaffold is quite complex and requires multistep chemical modification of the native keratin structure.

The first step of this process is the extraction of keratin performed in strong chemical conditions using thermochemical or microbial approaches [107]. When the extracted keratin is ready to be processed, it forms stable transparent films that show high biocompatibility and biostability, and that are able to induce cell adhesion in living rabbits [108]. Nevertheless, keratin cytocompatibility is debated as the role of keratin in hemostasis processes is not totally clear [109]. The most accepted hemostatic effect of keratin hydrogel is the formation of a wound seal by reticulated coagulated keratin that induces the formation of granulose tissue upon it.

Nonetheless, keratin hydrogels have been used as effective hemostatic solutions in rabbits affected by lethal liver injury [110]. In addition, several commercial keratin-based hemostatic products are available, including HemCon^®^ (Portland, OR, USA) and QuickClot^®^ (Morrisville, NC, USA).

Keratin and keratin hydrogels have also been used for the regeneration of peripheral nerves, as reported by Sierpinski et al. [111] using mice models. The authors used human keratin extracted from hair, improving Schwann cell proliferation, together with upregulation of their gene expression, improving neuronal functionality. Furthermore, the authors described enhanced axon regeneration in damaged tibial nerves. Similarly, Apel et al. [112] induced the acceleration of nerve regeneration using keratin hydrogels, improving axon density. Neat keratin and keratin hydrogels have been shown to induce neovascularization and inflammation with reduction in soft tissue adhesion on polymeric scaffolds [113]. The addition of nanostructured carbon improved the interfacial properties and magnified the mechanical and electrical performance [114].

Song et al. [115] used an electrospinning technique to produce a composite based on rGO, keratin and poly(caprolactone)(PCL) for the treatment of damaged wounds that intrinsically show poor self-healing capacity. The material produced was composed of fibers with an average diameter of 240 nm, which was able to enhance cell adhesion and proliferation, accelerating wound re-epithelialization. Interestingly, the formulation increased the potential of mitochondrial membranes, improving cell viability. Additionally, the authors observed an increase in moisture absorption and the permeability of the rGO scaffold, supporting the use of graphene-based keratin scaffolds for the treatment of skin wounds.

Considering the number of studies reported in the literature, graphene-containing keratin materials represent a new and not-entirely-mature technology, whereas the combination of fibrous keratin with CNT has been more extensively studied. Mahmoodi et al. [116] followed a protocol close to the one previous described for the production of a PCL-keratin matrix through electrospinning using carboxylated MWCNTs. The composites produced showed very thin fibers with an average diameter of up to 50 nm, with both the tensile strength and tensile modulus increased by up to 260% compared to neat PCL-keratin material. The CNT-based composite induced relevant enhancement of osteogenic differentiation in mesenchymal stem cells with significant mineralization on the scaffold surface.

Similarly, Asl et al. [117] replaced PCL with poly(hydroxybutyrate), reporting overexpression of alkaline phosphatase with a related increase in mineralization rate. The combination of keratin with nanostructured carbon appears to be a reliable route for the reduction of an inflammatory response to CNTs, neat keratin and graphene-like materials, together with improving both interfacial properties and bone tissue healing.

#### 4.1.3. Silk and Fibroin

Silk is a biopolymer composed mainly of two proteins, fibroin and sericin. Fibroin is a structural protein responsible for the strength and stiffness of silk, while sericin is a jelly protein that promotes adhesion of fibroin fibers together [118].

Fibroin is structured in long parallel β-sheet fibers interacting through hydrogen bonds. Fibroin is a hydrophobic amino-acid-rich material; its ordered folding process underlies the impressive mechanical properties of silk residues, which enable the protein to self-assemble into a stable, ordered structure [119]. As discussed by Salehi et al. [120], silk is highly biocompatible and its use in tissue repair is related to its morphology. Accordingly, thin silk fibers are currently used as surgical sutures, while porous silk represents a promising template for the production of scaffolds able to tune the conformation of silk [121].

Izyan et al. [122] evaluated the properties of graphene flakes dispersed into silk films, reporting an increase in β-sheet conformation, increasing both the crystallinity and the elongation at failure compared with neat silk materials. The modulation of the properties of silk can also be performed by directly acting on the feed of silk worms, as reported by Qu et al. [123]. The authors fed silkworms with mulberry leaves coated with 2 wt.% of GO, showing an increase in the elongation of recovered silk of up to 60% due to increase in α-helical regions. Furthermore, the authors reported that a reduction in the GO amount down to 1 wt.% promoted a selective increase in the Young’s modulus. Similar results were achieved by adding up to 1 wt.% of rGO [124]. The graphene-based silk composite can also be used as a template for the production of biocompatible, size-controlled pore hydrogels with good cell viability [125]. Wang et al. [126] also demonstrated that the direction of channels could be organized in the presence of GO-based silk materials.

There are three main production routes for graphene-like materials containing silk composites: the sol-gel approach [127], reverse phase casting [128] and electrospinning [129]. Reverse phase casting is used for the production of homogeneous hydrogel layers, while electrospinning enables creation of a proper fibrous mate. The sol-gel approach is used for the production of injectable and conformable silk scaffolds. Together with the flexibility in production routes, graphene-like-containing silk composites show remarkable biocompatibility and cell viability [124]. The use of such composites has found many applications in the production of biomaterials [130,131,132]. Pathmanapan and coworkers [133] dispersed GO into fibroin hydrogel as scaffolds for bone repair, showing enhanced levels of alkaline phosphatase activity. This approach can be boosted by adding hydroxyapatite to the mixture, promoting mesenchymal stem cell adhesion and proliferation with more effective bone repair [134]. Similar results were obtained by mixing GO-containing silk composites with poly(l-lactic-co-glycolic acid) up to 10 wt.%.

Furthermore, Eivazzadeh-Keihan et al. [135] tested GO-based silk composites mixed with alginate, demonstrating their compatibility with blood, reporting a hemolytic effect below 6% for a concentration of up to 1 mg/mL. Li et al. [136] moved a step forward by modifying GO-containing silk composites with tannic acid and strontium cations, eliminating any inflammatory response mediated by both cytokines and reactive oxygen species, for the treatment of osteoarthritis. Furthermore, the GO-based scaffolds promoted the formation of cartilage tissue in injured rat knees. However, the exploitation of the electrical conductivity of graphene and rGO has found a main application in the treatment of neuronal and nerve tissues. Magaz et al. [137] were able to regulate the neuronal cell response by using GO mixed with an rGO-containing silk microcomposite. The prepared material had a conductivity of up to 3 μS/m after hydration and was able to mimic the arrangement of the extracellular matrix. The authors used a neuronal cell line, reporting the extension of neurites by up to 250 μm after 5 days. Similar results were achieved using graphene flakes dispersed into spun silk [129,138], mat [139] or gel [140], though the dispersibility of graphene flakes remained challenging.

Colloidal composites based on GO and silk were used to repair myocardial tissue after infarction by mixing with growth factors [141].

CNTs can also be mixed with silk using electrospinning for the production of biocompatible composites with high electronic conductivity and improved mechanical properties [142], showing that, even when using a high loading of MWCNTs, the effect on silk fiber morphology remained negligible. Shrestha et al. [143] dispersed functionalized MWCNTs into silk mixed with poly(urethane) producing a highly conductive scaffold in which CNTs were aligned. The authors reported the proliferation of Schwann cells, together with the spontaneous outgrowth of neurites along the fiber direction. Additionally, injectable shape memory CNT-based silk composites were used to treat ischemic stroke, promoting the customized growth of new neuronal tissue [144].

### 4.2. Nanostructured Carbon-Containing Polysaccharide Composites

#### 4.2.1. Chitosan

Chitin is a functionalized polysaccharide containing units of β-(1,4)-N-acetyl-*d-*glucosamine produced as crystalline ordered microfibrils and is a crucial component in the formation of the cell walls of fungi, yeast and arthropod exoskeletons. Chitin has been widely used as an electrospun fiber mat by improving its mechanical properties by functionalization and coupling with polymers [145]. The as-produced chitin-derived fibers were shown to be biocompatible and able to promote both cell adhesion and proliferation without appreciable cytotoxicity.

Among chitin derivatives, chitosan is the most used and is produced by the simple partial deacetylation of neat chitin through alkaline or enzymatic hydrolysis [146]. Chitosan can be combined with GO, producing films with a low inflammatory response, advanced cicatrization, and good resorption in subdermal rat tissue [147]. As reported by Yilmaz et al. [148], chitosan combined with GO and hydroxyapatite showed a sponge-like morphology, together with both high cell viability and mechanical compressive strength, suggesting possible use as a bone replacement. This is supported also by research conducted by Depan et al. [149] in which GO-containing chitosan composites stimulated osteoblast growth. The authors suggested that osteoblast proliferation was due to both high water resorption and porous connection reducing their enzymatic degradation. Furthermore, the osteoblasts established intercellular connection mimicking real bone tissue. Liu et al. [150] demonstrated that GO-based chitosan composites with a lamellar conformation were able to promote the ordered growth of bone cell line MC3T3-E1 along the longitudinal direction. GO-based chitosan composites can simultaneously host toughening agents, such as hydroxyapatite [151] or another macrobiomolecule [152,153], to enhance its performance as a bone scaffold, without producing collateral reactions. Similar materials have been used for both cartilage [154,155] and dentary pulp [156] replacements. As reported by Fenge et al. [157], the unique weak and hydrogen-bond-type interactions occurring in GO-based chitosan composites presented a perfect case study of the realization of innovative wound dressing. The authors demonstrated that the reversible breakage–reformation of non-covalent interactions inside the composite allowed their adhesiveness to changing tissues, such as in the case of healing wounds. Additionally, GO-containing chitosan was shown to be able to stimulate nerve growth up to 20% more than neat chitosan scaffolds.

Similarly, Gupta et al. [158] dispersed both graphene nanoflakes and MWCNTs inside a chitosan matrix, promoting neurite outgrowth due to improved neural cell adhesion on the scaffold. CNT addition to a chitosan matrix modified the properties of chitosan hydrogels, improving the adhesion of several cell lines, as reported by Garnica-Palafox et al. [159]. CNT-based chitosan materials have also been used in bone tissue engineering due to improved cellular adhesion, together with high modulus and compressive strength, as reported by many authors [160,161,162], even without hydroxyapatite addition [163,164]. The superior mechanical properties of CNT-based chitosan represents a sound choice for cartilage replacement due to the high stress that this tissue can be exposed to. As reported by Mirmusavi et al. [165], PCL blended with chitosan and filled with 0.5 wt.% MWCNTs achieved a tensile strength of 34 MPa with high porosity and good compatibility with chondrocytes, enabling use in in vitro tests with good results.

#### 4.2.2. Hyaluronic Acid

Hyaluronic acid is a biopolymer composed of disaccharide units based on N-acetyl-D-glucosamine and glucuronic acid dimers with a molecular weight up to MDa; it can be found in the majority of biological tissues.

Hyaluronic acid use has been explored in viscosurgery for tissue protection during ophthalmological medical procedures, such as lens implantations or cataract surgery, and as a humor vitreous replacement [166].

Outside of surgical applications, hyaluronic acid can be used as a protective agent for wounds due to the moisturizing effect that accelerates tissue healing. Edmonds and coworkers [167] described the positive role of hyaluronic acid in the treatment of both wound infections and ulcers, preventing the formation of large scars [168].

Additionally, neat hyaluronic acid was used as a synovial fluid replacement and as an additive in cosmetic surgical treatments, inducing increase in the inter-tissue space with a filling effect, as reported by several studies [169,170].

Neat hyaluronic acid can promote the proliferation of chondrocytes, slowing down cartilage degradation and related ageing problems [171].

Furthermore, neat hyaluronic acid is able to reduce the inflammatory response by modulating cytokine metabolism, with a massive reduction in reactive oxygen species [172]. Neat hyaluronic acid carboxylic residues have been used for the production of quite stable hydrogels for the delivery of several types of active molecule, such as drugs, nucleic acids and antibodies [173]. Several authors [174,175] have described GO-based hyaluronic composites for osteogenic differentiation and drug release without causing degradative processes in relevant tissues [176].

Accordingly, Umar et al. [177] combined GO-based hyaluronic acid composite with bacterial cellulose for the production of a tough bone scaffold. The authors successfully produced a highly interconnected porous material able to inhibit bacterial growth and to promote osteocyte proliferation.

The addition of CNTs has created the possibility of producing hyaluronic composites enriched with pluripotent stem cells able to induce neurogenesis together with upregulation of calcium channels. CNT-containing hyaluronic fibers have been used for neural engineering [178]. The authors of this study investigated the stimulation of cultured neurons, showing that CNT-based composite stimulation prolonged growth, without suggesting a mechanism for this phenomenon.

#### 4.2.3. Cellulose

Cellulose is the most abundant polysaccharide and is composed of linear chains of glucose units connected to each other through a β 1-4 glycosidic chemical bond. Cellulose is a highly tunable template and can be shaped from nanocrystals to microfibrils based on an isolation methodology involving rearrangement of its hydrogen bond network. However, neat cellulose is not a good biological scaffold due to issues related to cellular interaction. Human cells are unable to adhere and to proliferate on neat cellulose films due to the hydrophilicity of the cellulose surface. This issue has been tackled by tailoring the cellulose hydroxyl residues by inserting species through simple chemical tailoring [179]. Cellulose tailoring is an easy and useful process able to produce a wide range of functionalized cellulose derivatives [180] used as biological scaffolds [181]. The most used tailoring processes are focused on the production of alkyl (i.e., methyl-, ethyl cellulose) or acetate derivatives [182].

Alternatively, neat cellulose can be blended with other polymers, as reported by Mao et al. [183]. The authors mixed neat cellulose with (poly)lactic acid filled with hydroxyapatite, demonstrating its effectiveness as a bone-repairing solution.

The main feature of nanostructured carbon-based cellulose composites is represented by the strong interaction between cellulose hydroxyl groups and the residual functionalities of carbon materials that tune cell adhesion, growth and proliferation [184].

Graphene and GO flakes dispersed into cellulose matrixes resulted in interesting properties, such as stimulation of osteogenesis [185,186], neural stem cell proliferation [187] and wound healing [188,189].

Similar results have been achieved using functionalized MCNTs, as reported by Khalid and co-workers [190].

## 5. Nanostructured Carbonaceous Materials and Related Biopolymer Composites for Tissue Engineering Applications: Weakness and Strengths

Nanostructured carbonaceous materials and related biopolymers represent an advanced means of addressing the many issues related to their use as neat materials. CNTs and graphene-related materials are the best candidates to improve both the mechanical and electrical properties of composites. However, several other solutions exist based on nanostructured carbon particles, which are particularly attractive for biomedical applications, such as carbon nano-onions [191]. This particular class of compounds can be used to produce drug delivery systems [192], and reinforce bulk [193] or film [194] materials, but the magnitude of improvement is still far from that achieved by adding CNTs or graphene-related materials. Opposite considerations apply when considering the use of the same nanostructured fillers combined with synthetic polymers, that show superior performance, such as ultra-high density poly(ethylene) (UHMWPE), as reported by Diabb Zavala et al. [195]. Here, the authors reported an increase in yield strength of up 65% of CNT-based UHMWPE composites, reaching a value beyond any biopolymer-based material. Nevertheless, UHMWPE and other olefins have issues related to their degradative routes due to friction and biological degradation [196,197], while biopolymers show higher biocompatibility with the production of less harmful degradation species [198,199].

A further important point to be considered is biopolymer isolation, that can represent a cost-determining step in biopolymer production. Similar considerations apply to the production of both CNTs and graphene materials that are still far from being large-scale and sufficiently standardized to meet biomedical industry requirements [200,201,202].

Nevertheless, the great economical value of biomedical solutions represents a driving force for rapidly achieving significant advancements in the large-scale industrial production of biopolymers, nanostructured carbon species and their composites.

This can lead to a new generation of multiresponsive materials useful for applications at the neural interface due to both their chemical and electrical properties [203,204]. Furthermore, new hybrid materials can open the way to the development of theranostic platforms based on electrical signal monitoring [205].

## 6. Conclusions

The use of biopolymers represents one of the most interesting frontiers in tissue engineering due to the great potential of biopolymer architectures naturally produced by the molecular engines of biological systems. Their combination with nanostructured carbon represents an additional step towards the production of complex and resilient species with enhanced electrical conductivity and mechanical properties, which will enable the creation of multifunctional composites for many applications in biological fields, such as regenerative medicine, scaffolds, and prosthesis production. Nonetheless, several issues remain unsolved, such as the high cost of biopolymers, CNTs and graphene and related materials, together with their difficult purification, processing, and standardization. Additionally, the degradative pathways of biopolymers are a key point to be considered for in vivo applications, particularly for prosthetic implants where they could represent a considerable advancement compared with the degradative pathways occurring with synthetic-polymer-based composites. Nevertheless, the full exploitation of nanostructured carbon-based biopolymer composites will surely lead to discoveries in many fields, such as regenerative medicine and neuronal implantation.

## References

[1] Guo L., Liang Z., Yang L., Du W., Yu T., Tang H., Li C., Qiu H. (2021). The role of natural polymers in bone tissue engineering. J. Control. Release.

[2] Ikada Y. (2006). Challenges in tissue engineering. J. R. Soc. Interface.

[3] Biswal T. (2021). Biopolymers for tissue engineering applications: A review. Mater. Today Proc..

[4] Marras E., Bartoli M., Tagliaferro A., Kanwar S., Kumar A., Nguyen T.A., Sharma S., Slimani Y. (2021). 16—Drug delivery. Biopolymeric Nanomaterials.

[5] Catania F., Bartoli M., Tagliaferro A. (2021). Biopolymer-nanoparticles hybrids. Biopolymeric Nanomaterials.

[6] Bendrea A.-D., Cianga L., Cianga I. (2011). Progress in the field of conducting polymers for tissue engineering applications. J. Biomater. Appl..

[7] Greil P. (2015). Perspectives of nano-carbon based engineering materials. Adv. Eng. Mater..

[8] Soni S.K., Thomas B., Kar V.R. (2020). A comprehensive review on CNTs and CNT-reinforced composites: Syntheses, characteristics and applications. Mater. Today Commun..

[9] Razaq A., Bibi F., Zheng X., Papadakis R., Jafri S.H.M., Li H. (2022). Review on graphene-, graphene oxide-, reduced graphene oxide-based flexible composites: From fabrication to applications. Materials.

[10] Amiryaghoubi N., Fathi M., Barzegari A., Barar J., Omidian H., Omidi Y. (2021). Recent advances in polymeric scaffolds containing carbon nanotube and graphene oxide for cartilage and bone regeneration. Mater. Today Commun..

[11] Ioniţă M., Vlăsceanu G.M., Watzlawek A.A., Voicu S.I., Burns J.S., Iovu H. (2017). Graphene and functionalized graphene: Extraordinary prospects for nanobiocomposite materials. Compos. Part B Eng..

[12] Mamidi N., Velasco Delgadillo R.M., Barrera E.V., Ramakrishna S., Annabi N. (2022). Carbonaceous nanomaterials incorporated biomaterials: The present and future of the flourishing field. Compos. Part B Eng..

[13] Nikolova M.P., Chavali M.S. (2019). Recent advances in biomaterials for 3D scaffolds: A review. Bioact. Mater..

[14] Chapekar M.S. (2000). Tissue engineering: Challenges and opportunities. J. Biomed. Mater. Res. Off. J. Soc. Biomater. Jpn. Soc. Biomater. Aust. Soc. Biomater. Korean Soc. Biomater..

[15] Bian W., Bursac N. (2008). Cellular/tissue engineering. IEEE Eng. Med. Biol. Mag..

[16] Reddy M.S.B., Ponnamma D., Choudhary R., Sadasivuni K.K. (2021). A comparative review of natural and synthetic biopolymer composite scaffolds. Polymers.

[17] Yannas I. (1975). Suppression of in vivo degradability and of immunogenicity of collagen by reaction with glycosaminoglycans. Polym. Prepr. Am. Chem. Soc..

[18] Yannas I., Burke J.F. (1980). Design of an artificial skin. I. Basic design principles. J. Biomed. Mater. Res..

[19] Patel N.R., Gohil P.P. (2012). A review on biomaterials: Scope, applications & human anatomy significance. Int. J. Emerg. Technol. Adv. Eng..

[20] Staudinger H. (2013). On Polymerization. A Source Book in Chemistry, 1900–1950.

[21] Kobayashi S., Sakamoto J., Kimura S. (2001). In vitro synthesis of cellulose and related polysaccharides. Prog. Polym. Sci..

[22] Raibaut L., El Mahdi O., Melnyk O. (2015). Solid phase protein chemical synthesis. Protein Ligation and Total Synthesis II.

[23] Bartoli M., Tagliaferro A. (2023). 25 Recent Polymers Advancements for Biomedical in Applications. Specialty Polymers: Fundamentals, Properties, Applications and Advances.

[24] Catania F., Marras E., Giorcelli M., Jagdale P., Lavagna L., Tagliaferro A., Bartoli M. (2021). A Review on Recent Advancements of Graphene and Graphene-Related Materials in Biological Applications. Appl. Sci..

[25] Yang W., Thordarson P., Gooding J.J., Ringer S.P., Braet F. (2007). Carbon nanotubes for biological and biomedical applications. Nanotechnology.

[26] Narsimha M., Hosam El-Din S., Said Moawad Mohamed E.-S. (2019). Cytotoxicity Evaluation of Carbon Nanotubes for Biomedical and Tissue Engineering Applications. Perspective of Carbon Nanotubes.

[27] Bussy C., Ali-Boucetta H., Kostarelos K. (2013). Safety Considerations for Graphene: Lessons Learnt from Carbon Nanotubes. Acc. Chem. Res..

[28] Edgar Jimenez-Cervantes A., Juventino L.B., Ana Laura M.H., Carlos V.S., Pramoda Kumar N. (2016). Graphene-Based Materials Functionalization with Natural Polymeric Biomolecules. Recent Advances in Graphene Research.

[29] Mintmire J.W., Dunlap B.I., White C.T. (1992). Are fullerene tubules metallic?. Phys. Rev. Lett..

[30] Yan J.-A., Ruan W., Chou M. (2009). Electron-phonon interactions for optical-phonon modes in few-layer graphene: First-principles calculations. Phys. Rev. B.

[31] Dresselhaus M., Jorio A., Saito R. (2010). Characterizing graphene, graphite, and carbon nanotubes by Raman spectroscopy. Annu. Rev. Condens. Matter Phys..

[32] Castro Neto A.H., Guinea F., Peres N.M.R., Novoselov K.S., Geim A.K. (2009). The electronic properties of graphene. Rev. Mod. Phys..

[33] Piatti E., Arbab A., Galanti F., Carey T., Anzi L., Spurling D., Roy A., Zhussupbekova A., Patel K.A., Kim J.M. (2021). Charge transport mechanisms in inkjet-printed thin-film transistors based on two-dimensional materials. Nat. Electron..

[34] Lee H.C., Liu W.-W., Chai S.-P., Mohamed A.R., Lai C.W., Khe C.-S., Voon C., Hashim U., Hidayah N. (2016). Synthesis of single-layer graphene: A review of recent development. Procedia Chem..

[35] Narita A., Wang X.-Y., Feng X., Müllen K. (2015). New advances in nanographene chemistry. Chem. Soc. Rev..

[36] Sun Z., Fang S., Hu Y.H. (2020). 3D Graphene Materials: From Understanding to Design and Synthesis Control. Chem. Rev..

[37] Piatti E., Galasso S., Tortello M., Nair J., Gerbaldi C., Bruna M., Borini S., Daghero D., Gonnelli R. (2017). Carrier mobility and scattering lifetime in electric double-layer gated few-layer graphene. Appl. Surf. Sci..

[38] Brisebois P., Siaj M. (2020). Harvesting graphene oxide–years 1859 to 2019: A review of its structure, synthesis, properties and exfoliation. J. Mater. Chem. C.

[39] Szabó T., Berkesi O., Forgó P., Josepovits K., Sanakis Y., Petridis D., Dékány I. (2006). Evolution of surface functional groups in a series of progressively oxidized graphite oxides. Chem. Mater..

[40] Lavagna L., Meligrana G., Gerbaldi C., Tagliaferro A., Bartoli M. (2020). Graphene and Lithium-Based Battery Electrodes: A Review of Recent Literature. Energies.

[41] Ahmed A., Singh A., Young S.-J., Gupta V., Singh M., Arya S. (2022). Synthesis Techniques and Advances in Sensing Applications of Reduced Graphene Oxide (rGO) Composites: A Review. Compos. Part A Appl. Sci. Manuf..

[42] Poh H.L., Šaněk F., Ambrosi A., Zhao G., Sofer Z., Pumera M. (2012). Graphenes prepared by Staudenmaier, Hofmann and Hummers methods with consequent thermal exfoliation exhibit very different electrochemical properties. Nanoscale.

[43] Lee X.J., Hiew B.Y.Z., Lai K.C., Lee L.Y., Gan S., Thangalazhy-Gopakumar S., Rigby S. (2019). Review on graphene and its derivatives: Synthesis methods and potential industrial implementation. J. Taiwan Inst. Chem. Eng..

[44] Kotchey G.P., Hasan S.A., Kapralov A.A., Ha S.H., Kim K., Shvedova A.A., Kagan V.E., Star A. (2012). A Natural Vanishing Act: The Enzyme-Catalyzed Degradation of Carbon Nanomaterials. Acc. Chem. Res..

[45] Yue H., Wei W., Yue Z., Wang B., Luo N., Gao Y., Ma D., Ma G., Su Z. (2012). The role of the lateral dimension of graphene oxide in the regulation of cellular responses. Biomaterials.

[46] Mu Q., Su G., Li L., Gilbertson B.O., Yu L.H., Zhang Q., Sun Y.-P., Yan B. (2012). Size-dependent cell uptake of protein-coated graphene oxide nanosheets. ACS Appl. Mater. Interfaces.

[47] Munir K.S., Wen C., Li Y. (2019). Carbon nanotubes and graphene as nanoreinforcements in metallic biomaterials: A review. Adv. Biosyst..

[48] Thompson B.C., Murray E., Wallace G.G. (2015). Graphite oxide to graphene. Biomaterials to bionics. Adv. Mater..

[49] Wang Q., Wang M., Wang K., Sun Y., Zhang H., Lu X., Duan K. (2020). Molecular mechanisms of interactions between BMP-2 and graphene: Effects of functional groups and microscopic morphology. Appl. Surf. Sci..

[50] Ni G., Wang Y., Wu X., Wang X., Chen S., Liu X. (2012). Graphene oxide absorbed anti-IL10R antibodies enhance LPS induced immune responses in vitro and in vivo. Immunol. Lett..

[51] Di Ianni E., Møller P., Vogel U.B., Jacobsen N.R. (2021). Pro-inflammatory response and genotoxicity caused by clay and graphene nanomaterials in A549 and THP-1 cells. Mutat. Res. Genet. Toxicol. Environ. Mutagen..

[52] Bahrami S., Solouk A., Mirzadeh H., Seifalian A.M. (2019). Electroconductive polyurethane/graphene nanocomposite for biomedical applications. Compos. Part B Eng..

[53] Pant H.R., Pokharel P., Joshi M.K., Adhikari S., Kim H.J., Park C.H., Kim C.S. (2015). Processing and characterization of electrospun graphene oxide/polyurethane composite nanofibers for stent coating. Chem. Eng. J..

[54] Belin T., Epron F. (2005). Characterization methods of carbon nanotubes: A review. Mater. Sci. Eng. B.

[55] Sanginario A., Miccoli B., Demarchi D. (2017). Carbon nanotubes as an effective opportunity for cancer diagnosis and treatment. Biosensors.

[56] Dresselhaus M., Dresselhaus G., Jorio A. (2004). Unusual properties and structure of carbon nanotubes. Annu. Rev. Mater. Res..

[57] Fagan J.A., Hároz E.H., Ihly R., Gui H., Blackburn J.L., Simpson J.R., Lam S., Hight Walker A.R., Doorn S.K., Zheng M. (2015). Isolation of >1 nm diameter single-wall carbon nanotube species using aqueous two-phase extraction. ACS Nano.

[58] Liu W.-W., Chai S.-P., Mohamed A.R., Hashim U. (2014). Synthesis and characterization of graphene and carbon nanotubes: A review on the past and recent developments. J. Ind. Eng. Chem..

[59] Navas H., Picher M., Andrieux-Ledier A., Fossard F., Michel T., Kozawa A., Maruyama T., Anglaret E., Loiseau A., Jourdain V. (2017). Unveiling the Evolutions of Nanotube Diameter Distribution during the Growth of Single-Walled Carbon Nanotubes. ACS Nano.

[60] Inam F., Reece M.J., Peijs T. (2011). Shortened carbon nanotubes and their influence on the electrical properties of polymer nanocomposites. J. Compos. Mater..

[61] Zhang R., Zhang Y., Zhang Q., Xie H., Qian W., Wei F. (2013). Growth of Half-Meter Long Carbon Nanotubes Based on Schulz–Flory Distribution. ACS Nano.

[62] Zhu Z., Wei N., Cheng W., Shen B., Sun S., Gao J., Wen Q., Zhang R., Xu J., Wang Y. (2019). Rate-selected growth of ultrapure semiconducting carbon nanotube arrays. Nat. Commun..

[63] Tasis D., Tagmatarchis N., Bianco A., Prato M. (2006). Chemistry of Carbon Nanotubes. Chem. Rev..

[64] Thess A., Lee R., Nikolaev P., Dai H., Petit P., Robert J., Xu C., Lee Y.H., Kim S.G., Rinzler A.G. (1996). Crystalline ropes of metallic carbon nanotubes. Science.

[65] Mansfield E., Feldman A., Chiaramonti A.N., Lehman J., Curtin A.E. (2015). Morphological and Electrical Characterization of MWCNT Papers and Pellets. J. Res. Natl. Inst. Stand. Technol..

[66] Han J. (2004). Structures and properties of carbon nanotubes. Carbon Nanotubes.

[67] Choi T.Y., Poulikakos D., Tharian J., Sennhauser U. (2005). Measurement of thermal conductivity of individual multiwalled carbon nanotubes by the 3-ω method. Appl. Phys. Lett..

[68] Cao J., Yan X., Xiao Y., Ding J. (2004). Thermal conductivity of zigzag single-walled carbon nanotubes: Role of the umklapp process. Phys. Rev. B.

[69] Fenoglio I., Greco G., Tomatis M., Muller J., Raymundo-Piñero E., Béguin F., Fonseca A., Nagy J.B., Lison D., Fubini B. (2008). Structural Defects Play a Major Role in the Acute Lung Toxicity of Multiwall Carbon Nanotubes: Physicochemical Aspects. Chem. Res. Toxicol..

[70] Muller J., Huaux F., Fonseca A., Nagy J.B., Moreau N., Delos M., Raymundo-Pinñero E., Béguin F., Kirsch-Volders M., Fenoglio I. (2008). Structural defects play a major role in the acute lung toxicity of multiwall carbon nanotubes: Toxicological aspects. Chem. Res. Toxicol..

[71] Visalli G., Facciolà A., Iannazzo D., Piperno A., Pistone A., Di Pietro A. (2017). The role of the iron catalyst in the toxicity of multi-walled carbon nanotubes (MWCNTs). J. Trace Elem. Med. Biol..

[72] Kostarelos K. (2008). The long and short of carbon nanotube toxicity. Nat. Biotechnol..

[73] Fenoglio I., Tomatis M., Lison D., Muller J., Fonseca A., Nagy J.B., Fubini B. (2006). Reactivity of carbon nanotubes: Free radical generation or scavenging activity?. Free Radic. Biol. Med..

[74] Huang B. (2020). Carbon nanotubes and their polymeric composites: The applications in tissue engineering. Biomanuf. Rev..

[75] Mamidi N., Leija H.M., Diabb J.M., Lopez Romo I., Hernandez D., Castrejón J.V., Martinez Romero O., Barrera E.V., Elias Zúñiga A. (2017). Cytotoxicity evaluation of unfunctionalized multiwall carbon nanotubes-ultrahigh molecular weight polyethylene nanocomposites. J. Biomed. Mater. Res. Part A.

[76] Vicentini N., Gatti T., Salerno M., Gomez Y.S.H., Bellon M., Gallio S., Marega C., Filippini F., Menna E. (2018). Effect of different functionalized carbon nanostructures as fillers on the physical properties of biocompatible poly (l-lactic acid) composites. Mater. Chem. Phys..

[77] Koppes A., Keating K., McGregor A., Koppes R., Kearns K., Ziemba A., McKay C., Zuidema J., Rivet C., Gilbert R. (2016). Robust neurite extension following exogenous electrical stimulation within single walled carbon nanotube-composite hydrogels. Acta Biomater..

[78] Sionkowska A., Skrzyński S., Śmiechowski K., Kołodziejczak A. (2017). The review of versatile application of collagen. Polym. Adv. Technol..

[79] Chattopadhyay S., Raines R.T. (2014). Collagen-based biomaterials for wound healing. Biopolymers.

[80] Shoulders M.D., Raines R.T. (2009). Collagen structure and stability. Annu. Rev. Biochem..

[81] Meek K.M., Boote C. (2004). The organization of collagen in the corneal stroma. Exp. Eye Res..

[82] Ferreira A.M., Gentile P., Chiono V., Ciardelli G. (2012). Collagen for bone tissue regeneration. Acta Biomater..

[83] Laurent G. (1987). Dynamic state of collagen: Pathways of collagen degradation in vivo and their possible role in regulation of collagen mass. Am. J. Physiol. Cell Physiol..

[84] Agarwal G., Agrawal A.K., Fatima A., Srivastava A. (2021). X-ray tomography analysis reveals the influence of graphene on porous morphology of collagen cryogels. Micron.

[85] Ilya Syafiqa Zulkifli N., Ibrahim N., Jaafar M., Teramoto N. (2022). The properties of the modified fish collagen peptide hydrogel. Mater. Today Proc..

[86] Girão A.F., Gonçalves G., Bhangra K.S., Phillips J.B., Knowles J., Irurueta G., Singh M.K., Bdkin I., Completo A., Marques P.A.A.P. (2016). Electrostatic self-assembled graphene oxide-collagen scaffolds towards a three-dimensional microenvironment for biomimetic applications. RSC Adv..

[87] Bahrami S., Baheiraei N., Shahrezaee M. (2021). Biomimetic reduced graphene oxide coated collagen scaffold for in situ bone regeneration. Sci. Rep..

[88] Liu S., Zhou C., Mou S., Li J., Zhou M., Zeng Y., Luo C., Sun J., Wang Z., Xu W. (2019). Biocompatible graphene oxide–collagen composite aerogel for enhanced stiffness and in situ bone regeneration. Mater. Sci. Eng. C.

[89] Chen Y., Zheng Z., Zhou R., Zhang H., Chen C., Xiong Z., Liu K., Wang X. (2019). Developing a Strontium-Releasing Graphene Oxide-/Collagen-Based Organic–Inorganic Nanobiocomposite for Large Bone Defect Regeneration via MAPK Signaling Pathway. ACS Appl. Mater. Interfaces.

[90] Zhou C., Luo C., Liu S., Jiang S., Liu X., Li J., Zhang X., Wu X., Sun J., Wang Z. (2022). Pearl-inspired graphene oxide-collagen microgel with multi-layer mineralization through microarray chips for bone defect repair. Mater. Today Bio.

[91] Zhou C., Liu S., Li J., Guo K., Yuan Q., Zhong A., Yang J., Wang J., Sun J., Wang Z. (2018). Collagen Functionalized With Graphene Oxide Enhanced Biomimetic Mineralization and in Situ Bone Defect Repair. ACS Appl. Mater. Interfaces.

[92] Lyu C., Cheng C., He Y., Qiu L., He Z., Zou D., Li D., Lu J. (2022). Graphene Hydrogel as a Porous Scaffold for Cartilage Regeneration. ACS Appl. Mater. Interfaces.

[93] Guo W., Wang S., Yu X., Qiu J., Li J., Tang W., Li Z., Mou X., Liu H., Wang Z. (2016). Construction of a 3D rGO–collagen hybrid scaffold for enhancement of the neural differentiation of mesenchymal stem cells. Nanoscale.

[94] Agarwal G., Kumar N., Srivastava A. (2021). Highly elastic, electroconductive, immunomodulatory graphene crosslinked collagen cryogel for spinal cord regeneration. Mater. Sci. Eng. C.

[95] Yılmaz E., Çakıroğlu B., Gökçe A., Findik F., Gulsoy H.O., Gulsoy N., Mutlu Ö., Özacar M. (2019). Novel hydroxyapatite/graphene oxide/collagen bioactive composite coating on Ti16Nb alloys by electrodeposition. Mater. Sci. Eng. C.

[96] Chen C.-Y., Tsai P.-H., Lin Y.-H., Huang C.-Y., Chung J.H.Y., Chen G.-Y. (2022). Controllable graphene oxide-based biocompatible hybrid interface as an anti-fibrotic coating for metallic implants. Mater. Today Bio.

[97] Liu J., Wang X., Saberi A., Heydari Z. (2023). The effect of Co-encapsulated GNPs-CNTs nanofillers on mechanical properties, degradation and antibacterial behavior of Mg-based composite. J. Mech. Behav. Biomed. Mater..

[98] Kim T., Sridharan I., Zhu B., Orgel J., Wang R. (2015). Effect of CNT on collagen fiber structure, stiffness assembly kinetics and stem cell differentiation. Mater. Sci. Eng. C.

[99] Lee J.H., Lee J.-Y., Yang S.H., Lee E.-J., Kim H.-W. (2014). Carbon nanotube–collagen three-dimensional culture of mesenchymal stem cells promotes expression of neural phenotypes and secretion of neurotrophic factors. Acta Biomater..

[100] Madhusoodan A.P., Das K., Mili B., Kumar K., Kumar A., Saxena A.C., Singh P., Dutt T., Bag S. (2019). In vitro proliferation and differentiation of canine bone marrow derived mesenchymal stem cells over hydroxyl functionalized CNT substrates. Biotechnol. Rep..

[101] Mao H., Kawazoe N., Chen G. (2013). Uptake and intracellular distribution of collagen-functionalized single-walled carbon nanotubes. Biomaterials.

[102] MacDonald R.A., Laurenzi B.F., Viswanathan G., Ajayan P.M., Stegemann J.P. (2005). Collagen–carbon nanotube composite materials as scaffolds in tissue engineering. J. Biomed. Mater. Res. Part A Off. J. Soc. Biomater. Jpn. Soc. Biomater. Aust. Soc. Biomater. Korean Soc. Biomater..

[103] Dong C., Lv Y. (2016). Application of collagen scaffold in tissue engineering: Recent advances and new perspectives. Polymers.

[104] Chi N., Wang R. (2018). Electrospun protein-CNT composite fibers and the application in fibroblast stimulation. Biochem. Biophys. Res. Commun..

[105] Ghosh S., Roy P., Lahiri D. (2022). Enhanced neurogenic differentiation on anisotropically conductive carbon nanotube reinforced polycaprolactone-collagen scaffold by applying direct coupling electrical stimulation. Int. J. Biol. Macromol..

[106] Yu H., Zhao H., Huang C., Du Y. (2017). Mechanically and Electrically Enhanced CNT–Collagen Hydrogels As Potential Scaffolds for Engineered Cardiac Constructs. ACS Biomater. Sci. Eng..

[107] Shavandi A., Silva T.H., Bekhit A.A., Bekhit A.E.-D.A. (2017). Keratin: Dissolution, extraction and biomedical application. Biomater. Sci..

[108] Borrelli M., Joepen N., Reichl S., Finis D., Schoppe M., Geerling G., Schrader S. (2015). Keratin films for ocular surface reconstruction: Evaluation of biocompatibility in an in-vivo model. Biomaterials.

[109] Rahmany M.B., Hantgan R.R., Van Dyke M. (2013). A mechanistic investigation of the effect of keratin-based hemostatic agents on coagulation. Biomaterials.

[110] Aboushwareb T., Eberli D., Ward C., Broda C., Holcomb J., Atala A., Van Dyke M. (2009). A keratin biomaterial gel hemostat derived from human hair: Evaluation in a rabbit model of lethal liver injury. J. Biomed. Mater. Res. Part B Appl. Biomater..

[111] Sierpinski P., Garrett J., Ma J., Apel P., Klorig D., Smith T., Koman L.A., Atala A., Van Dyke M. (2008). The use of keratin biomaterials derived from human hair for the promotion of rapid regeneration of peripheral nerves. Biomaterials.

[112] Apel P.J., Garrett J.P., Sierpinski P., Ma J., Atala A., Smith T.L., Koman L.A., Van Dyke M.E. (2008). Peripheral nerve regeneration using a keratin-based scaffold: Long-term functional and histological outcomes in a mouse model. J. Hand Surg..

[113] Loschke F., Seltmann K., Bouameur J.-E., Magin T.M. (2015). Regulation of keratin network organization. Curr. Opin. Cell Biol..

[114] Li Y.B., Liu H.H., Wang X.C., Zhang X.X. (2019). Fabrication and performance of wool keratin–functionalized graphene oxide composite fibers. Mater. Today Sustain..

[115] Song Z., Wang J., Tan S., Gao J., Wang L. (2023). Conductive biomimetic bilayer fibrous scaffold for skin regeneration. Colloids Surf. A Physicochem. Eng. Asp..

[116] Mahmoodi M., Haghighi V., Mirhaj M., Tavafoghi M., Shams F., Darabi A. (2021). Highly osteogenic and mechanically strong nanofibrous scaffolds based on functionalized multi-walled carbon nanotubes-reinforced electrospun keratin/poly(ε-caprolactone). Mater. Today Commun..

[117] Asl M.A., Karbasi S., Beigi-Boroujeni S., Benisi S.Z., Saeed M. (2022). Polyhydroxybutyrate-starch/carbon nanotube electrospun nanocomposite: A highly potential scaffold for bone tissue engineering applications. Int. J. Biol. Macromol..

[118] Liu B., Song Y.-W., Jin L., Wang Z.-J., Pu D.-Y., Lin S.-Q., Zhou C., You H.-J., Ma Y., Li J.-M. (2015). Silk structure and degradation. Colloids Surf. B Biointerfaces.

[119] Römer L., Scheibel T. (2008). The elaborate structure of spider silk: Structure and function of a natural high performance fiber. Prion.

[120] Salehi S., Koeck K., Scheibel T. (2020). Spider silk for tissue engineering applications. Molecules.

[121] Altman G.H., Diaz F., Jakuba C., Calabro T., Horan R.L., Chen J., Lu H., Richmond J., Kaplan D.L. (2003). Silk-based biomaterials. Biomaterials.

[122] Izyan Syazana Mohd Yusoff N., Uzir Wahit M., Jaafar J., Wong T.-W. (2018). Characterization of Graphene-Silk Fibroin Composites Film. Mater. Today: Proc..

[123] Qu J., Dai M., Ye W., Fang Y., Bian D., Su W., Li F., Sun H., Wei J., Li B. (2021). Study on the effect of graphene oxide (GO) feeding on silk properties based on segmented precise measurement. J. Mech. Behav. Biomed. Mater..

[124] Nalvuran H., Elçin A.E., Elçin Y.M. (2018). Nanofibrous silk fibroin/reduced graphene oxide scaffolds for tissue engineering and cell culture applications. Int. J. Biol. Macromol..

[125] Dorishetty P., Balu R., Gelmi A., Mata J.P., Quigley A., Dutta N.K., Choudhury N.R. (2022). Microporosity engineered printable silk/graphene hydrogels and their cytocompatibility evaluations. Mater. Today Adv..

[126] Wang L., Lian J., Xia Y., Guo Y., Xu C., Zhang Y., Xu J., Zhang X., Li B., Zhao B. (2022). A study on in vitro and in vivo bioactivity of silk fibroin/nano-hydroxyapatite/graphene oxide composite scaffolds with directional channels. Colloids Surf. A Physicochem. Eng. Asp..

[127] Wang L., Lu R., Hou J., Nan X., Xia Y., Guo Y., Meng K., Xu C., Wang X., Zhao B. (2020). Application of injectable silk fibroin/graphene oxide hydrogel combined with bone marrow mesenchymal stem cells in bone tissue engineering. Colloids Surf. A Physicochem. Eng. Asp..

[128] Wang S.-D., Ma Q., Wang K., Ma P.-B. (2018). Strong and biocompatible three-dimensional porous silk fibroin/graphene oxide scaffold prepared by phase separation. Int. J. Biol. Macromol..

[129] Aznar-Cervantes S., Pagán A., Martínez J.G., Bernabeu-Esclapez A., Otero T.F., Meseguer-Olmo L., Paredes J.I., Cenis J.L. (2017). Electrospun silk fibroin scaffolds coated with reduced graphene promote neurite outgrowth of PC-12 cells under electrical stimulation. Mater. Sci. Eng. C.

[130] Xu Z., Ma Y., Dai H., Tan S., Han B. (2022). Advancements and Applications in the Composites of Silk Fibroin and Graphene-Based Materials. Polymers.

[131] López Barreiro D., Yeo J., Tarakanova A., Martin-Martinez F.J., Buehler M.J. (2019). Multiscale Modeling of Silk and Silk-Based Biomaterials—A Review. Macromol. Biosci..

[132] Sun J., Shakya S., Gong M., Liu G., Wu S., Xiang Z. (2019). Combined application of graphene-family materials and silk fibroin in biomedicine. ChemistrySelect.

[133] Pathmanapan S., Periyathambi P., Anandasadagopan S.K. (2020). Fibrin hydrogel incorporated with graphene oxide functionalized nanocomposite scaffolds for bone repair—In vitro and in vivo study. Nanomed. Nanotechnol. Biol. Med..

[134] Wang Q., Chu Y., He J., Shao W., Zhou Y., Qi K., Wang L., Cui S. (2017). A graded graphene oxide-hydroxyapatite/silk fibroin biomimetic scaffold for bone tissue engineering. Mater. Sci. Eng. C.

[135] Eivazzadeh-Keihan R., Radinekiyan F., Madanchi H., Aliabadi H.A.M., Maleki A. (2020). Graphene oxide/alginate/silk fibroin composite as a novel bionanostructure with improved blood compatibility, less toxicity and enhanced mechanical properties. Carbohydr. Polym..

[136] Li Y., Chen M., Yan J., Zhou W., Gao S., Liu S., Li Q., Zheng Y., Cheng Y., Guo Q. (2021). Tannic acid/Sr^2+^-coated silk/graphene oxide-based meniscus scaffold with anti-inflammatory and anti-ROS functions for cartilage protection and delaying osteoarthritis. Acta Biomater..

[137] Magaz A., Li X., Gough J.E., Blaker J.J. (2021). Graphene oxide and electroactive reduced graphene oxide-based composite fibrous scaffolds for engineering excitable nerve tissue. Mater. Sci. Eng. C.

[138] Zhang C., Fan S., Shao H., Hu X., Zhu B., Zhang Y. (2019). Graphene trapped silk scaffolds integrate high conductivity and stability. Carbon.

[139] Zhang C., Wang X., Fan S., Lan P., Cao C., Zhang Y. (2021). Silk fibroin/reduced graphene oxide composite mats with enhanced mechanical properties and conductivity for tissue engineering. Colloids Surf. B Biointerfaces.

[140] Wang L., Song D., Zhang X., Ding Z., Kong X., Lu Q., Kaplan D.L. (2019). Silk–Graphene Hybrid Hydrogels with Multiple Cues to Induce Nerve Cell Behavior. ACS Biomater. Sci. Eng..

[141] Yuan Z., Qin Q., Yuan M., Wang H., Li R. (2020). Development and novel design of clustery graphene oxide formed Conductive Silk hydrogel cell vesicle to repair and routine care of myocardial infarction: Investigation of its biological activity for cell delivery applications. J. Drug Deliv. Sci. Technol..

[142] Gandhi M., Yang H., Shor L., Ko F. (2009). Post-spinning modification of electrospun nanofiber nanocomposite from Bombyx mori silk and carbon nanotubes. Polymer.

[143] Shrestha S., Shrestha B.K., Lee J., Joong O.K., Kim B.-S., Park C.H., Kim C.S. (2019). A conducting neural interface of polyurethane/silk-functionalized multiwall carbon nanotubes with enhanced mechanical strength for neuroregeneration. Mater. Sci. Eng. C.

[144] Wang J., Li X., Song Y., Su Q., Xiaohalati X., Yang W., Xu L., Cai B., Wang G., Wang Z. (2021). Injectable silk sericin scaffolds with programmable shape-memory property and neuro-differentiation-promoting activity for individualized brain repair of severe ischemic stroke. Bioact. Mater..

[145] Shalumon K., Binulal N., Selvamurugan N., Nair S., Menon D., Furuike T., Tamura H., Jayakumar R. (2009). Electrospinning of carboxymethyl chitin/poly (vinyl alcohol) nanofibrous scaffolds for tissue engineering applications. Carbohydr. Polym..

[146] Elieh-Ali-Komi D., Hamblin M.R. (2016). Chitin and chitosan: Production and application of versatile biomedical nanomaterials. Int. J. Adv. Res..

[147] Valencia A.M., Valencia C.H., Zuluaga F., Grande-Tovar C.D. (2021). Synthesis and fabrication of films including graphene oxide functionalized with chitosan for regenerative medicine applications. Heliyon.

[148] Yılmaz P., Öztürk Er E., Bakırdere S., Ülgen K., Özbek B. (2019). Application of supercritical gel drying method on fabrication of mechanically improved and biologically safe three-component scaffold composed of graphene oxide/chitosan/hydroxyapatite and characterization studies. J. Mater. Res. Technol..

[149] Depan D., Girase B., Shah J.S., Misra R.D.K. (2011). Structure–process–property relationship of the polar graphene oxide-mediated cellular response and stimulated growth of osteoblasts on hybrid chitosan network structure nanocomposite scaffolds. Acta Biomater..

[150] Liu Y., Fang N., Liu B., Song L., Wen B., Yang D. (2018). Aligned porous chitosan/graphene oxide scaffold for bone tissue engineering. Mater. Lett..

[151] Prakash J., Prema D., Venkataprasanna K.S., Balagangadharan K., Selvamurugan N., Venkatasubbu G.D. (2020). Nanocomposite chitosan film containing graphene oxide/hydroxyapatite/gold for bone tissue engineering. Int. J. Biol. Macromol..

[152] Sivashankari P.R., Prabaharan M. (2020). Three-dimensional porous scaffolds based on agarose/chitosan/graphene oxide composite for tissue engineering. Int. J. Biol. Macromol..

[153] Souza A.P.C., Neves J.G., Navarro da Rocha D., Lopes C.C., Moraes Â.M., Correr-Sobrinho L., Correr A.B. (2022). Chitosan/Xanthan membrane containing hydroxyapatite/Graphene oxide nanocomposite for guided bone regeneration. J. Mech. Behav. Biomed. Mater..

[154] Shamekhi M.A., Mirzadeh H., Mahdavi H., Rabiee A., Mohebbi-Kalhori D., Baghaban Eslaminejad M. (2019). Graphene oxide containing chitosan scaffolds for cartilage tissue engineering. Int. J. Biol. Macromol..

[155] Cao L., Zhang F., Wang Q., Wu X. (2017). Fabrication of chitosan/graphene oxide polymer nanofiber and its biocompatibility for cartilage tissue engineering. Mater. Sci. Eng. C.

[156] Amiryaghoubi N., Noroozi Pesyan N., Fathi M., Omidi Y. (2020). Injectable thermosensitive hybrid hydrogel containing graphene oxide and chitosan as dental pulp stem cells scaffold for bone tissue engineering. Int. J. Biol. Macromol..

[157] Feng W., Wang Z. (2022). Shear-thinning and self-healing chitosan-graphene oxide hydrogel for hemostasis and wound healing. Carbohydr. Polym..

[158] Gupta P., Agrawal A., Murali K., Varshney R., Beniwal S., Manhas S., Roy P., Lahiri D. (2019). Differential neural cell adhesion and neurite outgrowth on carbon nanotube and graphene reinforced polymeric scaffolds. Mater. Sci. Eng. C.

[159] Garnica-Palafox I.M., Estrella-Monroy H.O., Vázquez-Torres N.A., Álvarez-Camacho M., Castell-Rodríguez A.E., Sánchez-Arévalo F.M. (2020). Influence of multi-walled carbon nanotubes on the physico-chemical and biological responses of chitosan-based hybrid hydrogels. Carbohydr. Polym..

[160] Ali A., Bano S., Priyadarshi R., Negi Y.S. (2019). Effect of carbon based fillers on properties of Chitosan/PVA/βTCP based composite scaffold for bone tissue engineering. Mater. Today Proc..

[161] Venkatesan J., Qian Z.-J., Ryu B., Ashok Kumar N., Kim S.-K. (2011). Preparation and characterization of carbon nanotube-grafted-chitosan—Natural hydroxyapatite composite for bone tissue engineering. Carbohydr. Polym..

[162] Türk S., Altınsoy I., Çelebi Efe G., Ipek M., Özacar M., Bindal C. (2018). 3D porous collagen/functionalized multiwalled carbon nanotube/chitosan/hydroxyapatite composite scaffolds for bone tissue engineering. Mater. Sci. Eng. C.

[163] Venkatesan J., Ryu B., Sudha P.N., Kim S.-K. (2012). Preparation and characterization of chitosan–carbon nanotube scaffolds for bone tissue engineering. Int. J. Biol. Macromol..

[164] Gholizadeh S., Moztarzadeh F., Haghighipour N., Ghazizadeh L., Baghbani F., Shokrgozar M.A., Allahyari Z. (2017). Preparation and characterization of novel functionalized multiwalled carbon nanotubes/chitosan/β-Glycerophosphate scaffolds for bone tissue engineering. Int. J. Biol. Macromol..

[165] Mirmusavi M.H., Ahmadian M., Karbasi S. (2022). Polycaprolactone-chitosan/multi-walled carbon nanotube: A highly strengthened electrospun nanocomposite scaffold for cartilage tissue engineering. Int. J. Biol. Macromol..

[166] Kretz F.T., Limberger I.-J., Auffarth G.U. (2014). Corneal endothelial cell coating during phacoemulsification using a new dispersive hyaluronic acid ophthalmic viscosurgical device. J. Cataract Refract. Surg..

[167] Edmonds M., Bates M., Doxford M., Gough A., Foster A. (2000). New treatments in ulcer healing and wound infection. Diabetes Metab. Res. Rev..

[168] Namazi M.R., Fallahzadeh M.K., Schwartz R.A. (2011). Strategies for prevention of scars: What can we learn from fetal skin?. Int. J. Dermatol..

[169] Monheit G.D., Coleman K.M. (2006). Hyaluronic acid fillers. Dermatol. Ther..

[170] Chhetri D.K., Mendelsohn A.H. (2010). Hyaluronic acid for the treatment of vocal fold scars. Curr. Opin. Otolaryngol. Head Neck Surg..

[171] Ishida O., Tanaka Y., Morimoto I., Takigawa M., Eto S. (1997). Chondrocytes are regulated by cellular adhesion through CD44 and hyaluronic acid pathway. J. Bone Miner. Res..

[172] Gotoh S., Onaya J., Abe M., Miyazaki K., Hamai A., Horie K., Tokuyasu K. (1993). Effects of the molecular weight of hyaluronic acid and its action mechanisms on experimental joint pain in rats. Ann. Rheum. Dis..

[173] Huang G., Huang H. (2018). Application of hyaluronic acid as carriers in drug delivery. Drug Deliv..

[174] Lee S.J., Nah H., Heo D.N., Kim K.-H., Seok J.M., Heo M., Moon H.-J., Lee D., Lee J.S., An S.Y. (2020). Induction of osteogenic differentiation in a rat calvarial bone defect model using an In situ forming graphene oxide incorporated glycol chitosan/oxidized hyaluronic acid injectable hydrogel. Carbon.

[175] Rajan Unnithan A., Ramachandra Kurup Sasikala A., Park C.H., Kim C.S. (2017). A unique scaffold for bone tissue engineering: An osteogenic combination of graphene oxide–hyaluronic acid–chitosan with simvastatin. J. Ind. Eng. Chem..

[176] Patil R., Kansara V., Ray D., Aswal V.K., Jha P.K., Bahadur P., Tiwari S. (2019). Slow degrading hyaluronic acid hydrogel reinforced with cationized graphene nanosheets. Int. J. Biol. Macromol..

[177] Umar Aslam Khan M., Haider S., Haider A., Izwan Abd Razak S., Rafiq Abdul Kadir M., Shah S.A., Javed A., Shakir I., Al-Zahrani A.A. (2021). Development of porous, antibacterial and biocompatible GO/n-HAp/bacterial cellulose/β-glucan biocomposite scaffold for bone tissue engineering. Arab. J. Chem..

[178] Steel E.M., Azar J.-Y., Sundararaghavan H.G. (2020). Electrospun hyaluronic acid-carbon nanotube nanofibers for neural engineering. Materialia.

[179] Kalia S., Boufi S., Celli A., Kango S. (2014). Nanofibrillated cellulose: Surface modification and potential applications. Colloid Polym. Sci..

[180] Jedvert K., Heinze T. (2017). Cellulose modification and shaping—A review. J. Polym. Eng..

[181] Seddiqi H., Oliaei E., Honarkar H., Jin J., Geonzon L.C., Bacabac R.G., Klein-Nulend J. (2021). Cellulose and its derivatives: Towards biomedical applications. Cellulose.

[182] Tavakolian M., Jafari S.M., van de Ven T.G. (2020). A review on surface-functionalized cellulosic nanostructures as biocompatible antibacterial materials. Nano Micro Lett..

[183] Mao D., Li Q., Bai N., Dong H., Li D. (2018). Porous stable poly(lactic acid)/ethyl cellulose/hydroxyapatite composite scaffolds prepared by a combined method for bone regeneration. Carbohydr. Polym..

[184] Luo H., Ao H., Peng M., Yao F., Yang Z., Wan Y. (2019). Effect of highly dispersed graphene and graphene oxide in 3D nanofibrous bacterial cellulose scaffold on cell responses: A comparative study. Mater. Chem. Phys..

[185] Li J., Liu X., Crook J.M., Wallace G.G. (2020). Electrical stimulation-induced osteogenesis of human adipose derived stem cells using a conductive graphene-cellulose scaffold. Mater. Sci. Eng. C.

[186] Liu X., Shen H., Song S., Chen W., Zhang Z. (2017). Accelerated biomineralization of graphene oxide-incorporated cellulose acetate nanofibrous scaffolds for mesenchymal stem cell osteogenesis. Colloids Surf. B Biointerfaces.

[187] Guo R., Li J., Chen C., Xiao M., Liao M., Hu Y., Liu Y., Li D., Zou J., Sun D. (2021). Biomimetic 3D bacterial cellulose-graphene foam hybrid scaffold regulates neural stem cell proliferation and differentiation. Colloids Surf. B Biointerfaces.

[188] Aly A.A., Ahmed M.K. (2021). Nanofibers of cellulose acetate containing ZnO nanoparticles/graphene oxide for wound healing applications. Int. J. Pharm..

[189] Soliman M., Sadek A.A., Abdelhamid H.N., Hussein K. (2021). Graphene oxide-cellulose nanocomposite accelerates skin wound healing. Res. Vet. Sci..

[190] Khalid A., Madni A., Raza B., Islam M.U., Hassan A., Ahmad F., Ali H., Khan T., Wahid F. (2022). Multiwalled carbon nanotubes functionalized bacterial cellulose as an efficient healing material for diabetic wounds. Int. J. Biol. Macromol..

[191] Mamidi N., Delgadillo R.M.V., González-Ortiz A. (2021). Engineering of carbon nano-onion bioconjugates for biomedical applications. Mater. Sci. Eng. C.

[192] Mamidi N., González-Ortiz A., Lopez Romo I., Barrera E.V. (2019). Development of Functionalized Carbon Nano-Onions Reinforced Zein Protein Hydrogel Interfaces for Controlled Drug Release. Pharmaceutics.

[193] Mamidi N., Villela Castrejón J., González-Ortiz A. (2020). Rational design and engineering of carbon nano-onions reinforced natural protein nanocomposite hydrogels for biomedical applications. J. Mech. Behav. Biomed. Mater..

[194] Mamidi N., Velasco Delgadillo R.M., Gonzáles Ortiz A., Barrera E.V. (2020). Carbon Nano-Onions Reinforced Multilayered Thin Film System for Stimuli-Responsive Drug Release. Pharmaceutics.

[195] Diabb Zavala J.M., Leija Gutiérrez H.M., Segura-Cárdenas E., Mamidi N., Morales-Avalos R., Villela-Castrejón J., Elías-Zúñiga A. (2021). Manufacture and mechanical properties of knee implants using SWCNTs/UHMWPE composites. J. Mech. Behav. Biomed. Mater..

[196] Choudhury D., Ranuša M., Fleming R.A., Vrbka M., Křupka I., Teeter M.G., Goss J., Zou M. (2018). Mechanical wear and oxidative degradation analysis of retrieved ultra high molecular weight polyethylene acetabular cups. J. Mech. Behav. Biomed. Mater..

[197] Diabb J., Juarez-Hernandez A., Reyes A., González-Rivera C., Hernandez-Rodriguez M. (2009). Failure analysis for degradation of a polyethylene knee prosthesis component. Eng. Fail. Anal..

[198] Sell S.A., McClure M.J., Garg K., Wolfe P.S., Bowlin G.L. (2009). Electrospinning of collagen/biopolymers for regenerative medicine and cardiovascular tissue engineering. Adv. Drug Deliv. Rev..

[199] Rebelo R., Fernandes M., Fangueiro R. (2017). Biopolymers in medical implants: A brief review. Procedia Eng..

[200] Paradise M., Goswami T. (2007). Carbon nanotubes—production and industrial applications. Mater. Des..

[201] Phiri J., Gane P., Maloney T.C. (2017). General overview of graphene: Production, properties and application in polymer composites. Mater. Sci. Eng. B.

[202] Kauling A.P., Seefeldt A.T., Pisoni D.P., Pradeep R.C., Bentini R., Oliveira R.V., Novoselov K.S., Castro Neto A.H. (2018). The worldwide graphene flake production. Adv. Mater..

[203] Guo Y., Jiang S., Grena B.J., Kimbrough I.F., Thompson E.G., Fink Y., Sontheimer H., Yoshinobu T., Jia X. (2017). Polymer composite with carbon nanofibers aligned during thermal drawing as a microelectrode for chronic neural interfaces. Acs Nano.

[204] Fattahi P., Yang G., Kim G., Abidian M.R. (2014). A review of organic and inorganic biomaterials for neural interfaces. Adv. Mater..

[205] Kim M., Jang J., Cha C. (2017). Carbon nanomaterials as versatile platforms for theranostic applications. Drug Discov. Today.

